# Supplementary material for: Learning curve and surgical outcome of robotic assisted colorectal surgery with ERAS program
Source: Sci Rep. 2022 Nov 29;12:20566. doi: 10.1038/s41598-022-24665-w (PMC9709162; doi:10.1038/s41598-022-24665-w)
Supplement: Supplementary file 2 — Supplementary Information 2. [file 41598_2022_24665_MOESM2_ESM.zip › RAL Raw data-Ñ[▒K/127/Patho_ñw╝╨░O▒Kñσ.pdf]

臺中榮民總醫院 臺中市臺灣大道四段1650號 病理部活體組織切片 臨床醫學實驗及病理診斷中心 檢驗(查)報告

[redacted]

Pathologic diagnosis:
1. Sigmoid colon, robotic assisted anterior resection --- Lipoma. The bilateral cut ends and mesocolic lymph nodes (0/16) are unremarkable.
2. Sigmoid colon, distal cut end, resection --- Unremarkable.

Gross description:
The specimen consists of 1) a segment of colon, 12 cm in length and 5.5 cm in proximal cut end circumference, 5.5 cm in distal circumference, and mesocolic soft tissue. A rounded and pedunculated tumor, 3.3x3x3 cm, is noted, 3.5 cm to the distal cut end. On cross section, the tumor mainly located in submucosa with 10 cm to the radial margin. The cut surfaces show homogeneous yellow soft adipose tissue. The both cut ends are free of tumor grossly. 2) distal cut end, a piece of tan mucosa-covered soft tissue, 2x2x1 cm. Representative part for section: A) proximal cut end B) distal cut end C-F) tumor G) colon H) radial margin J) specimen 2 K-L) lymph nodes.
#T-59300_2 #M-88500_2 0 1105 000000
#T-C4560_2 #M-09183_2 0 1105 000000

已電子簽章

敬告：本單僅供醫師參考使用，不具任何申請效力！！
請病患妥為保管，若不慎遺失需自負個人資料外洩責任！
